# Association of prognostic nutritional index with long-term mortality in patients receiving percutaneous coronary intervention for acute coronary syndrome: a meta-analysis

**DOI:** 10.1038/s41598-023-40312-4

**Published:** 2023-08-11

**Authors:** Wei-Ting Chang, Cheuk-Kwan Sun, Jheng-Yan Wu, Chia-Hung Yu, Ying-Jen Chang, Ming-Chung Lin, Kuo-Mao Lan, I-Wen Chen, Kuo-Chuan Hung

**Affiliations:** 1 Division of Cardiology, Department of Internal Medicine, Chi-Mei Medical Center, Tainan City, Taiwan; 2 Department of Biotechnology, Southern Taiwan University of Science and Technology, Tainan City, Taiwan; 3 School of Medicine and Doctoral Program of Clinical and Experimental Medicine, College of Medicine and Center of Excellence for Metabolic Associated Fatty Liver Disease, National Sun Yat-sen University, Kaohsiung City, Taiwan; 4 Department of Emergency Medicine, E-Da Dachang Hospital, I-Shou University, Kaohsiung City, Taiwan; 5 School of Medicine for International Students, College of Medicine, I-Shou University, Kaohsiung City, Taiwan; 6 Department of Nutrition, Chi Mei Medical Center, Tainan City, Taiwan; 7 Department of Anesthesiology, Chi Mei Medical Center, No. 901, ChungHwa Road, YungKung Dist, Tainan City, 71004 Taiwan; 8 Department of Anesthesiology, Chi Mei Medical Center, Liouying, Tainan City, Taiwan; 9 School of Medicine, College of Medicine, National Sun Yat-Sen University, Kaohsiung City, Taiwan

**Keywords:** Biomarkers, Cardiology, Risk factors

## Abstract

The predictive value of the prognostic nutritional index (PNI) for the long-term prognosis of patients with acute coronary syndrome (ACS) remains uncertain. Medline, Embase, Cochrane Library, and Google Scholar were searched from inception until January 2023 to study the relationship between all-cause mortality risk and PNI in patients receiving percutaneous coronary intervention for ACS (i.e., primary outcome). Thirteen observational studies were included in this meta-analysis. Analysis of seven studies using PNI as a categorical variable showed a pooled hazard ratio (HR) of all-cause mortality of 2.97 (95% CI 1.65 to 5.34, *p* = 0.0003, I^2^ = 89%, n = 11,245) for patients with a low PNI. The meta-analysis also showed a higher risk of major adverse cardiovascular events (MACEs) in patients with a low PNI (HR 2.04; 95% CI 1.59 to 2.61; *p* < 0.00001; I^2^ = 21%; n = 8534). Moreover, advanced age, diabetes mellitus, and high Global Registry of Acute Coronary Events risk scores were associated with a high risk of all-cause mortality, whereas a high body mass index was associated with a low risk of all-cause mortality. The results showed an association between a low PNI and an increased risk of long-term mortality in patients undergoing coronary interventions for ACS. Further randomized controlled trials are necessary to confirm these findings.

## Introduction

Despite the dramatic reduction in cardiovascular disease-related mortality over the last few decades, ischemic heart disease continues to be the leading cause of death worldwide^1^, and significantly impairs the quality of life of its survivors^2^. Although improvements in interventional and antithrombotic treatments, secondary prevention, and risk factor modification during the past decade have decreased the short-term mortality rate, such as in-hospital and 30 days mortality after myocardial infarction, to approximately 5–8% after myocardial infarction^1,3^, the relatively high long-term mortality (i.e., 10–12%)^1,4^ highlights the need to identify high-risk patients and modify the risk factors at an early stage. Malnutrition, which is commonly observed in patients with acute coronary syndrome (ACS), can lead to reduced immune function and increased inflammation, which negatively impacts disease outcomes and increases the risk of complications^5,6^. Indeed, poor nutritional status has been identified as an independent risk factor for the development and progression of ACS^5,7^. Therefore, recognizing and addressing the issue of malnutrition in patients with ACS is of paramount importance in enhancing overall patient outcomes and alleviating the burden of complications.

The Prognostic Nutritional Index (PNI), which combines two nutritional markers, namely serum albumin and total lymphocyte count, has been validated in evaluating the nutritional and inflammatory status of critically ill patients to predict outcomes such as the length of hospital stay, postoperative delirium, and mortality and morbidity in cancer and non-cancer surgical settings^8–13^. Although a low PNI score (i.e., poor nutritional status) has been shown to be related to increased morbidity and mortality in patients with malignancy, its role as an indicator of the long-term outcome in patients receiving coronary interventions for ACS remains controversial^6,7,14–16^. In a recent study, Chen et al. demonstrated the potential benefits of incorporating PNI into the Global Registry of Acute Coronary Events (GRACE) score for the prediction of long-term all-cause mortality in patients diagnosed with ACS^16^. In contrast, Basta et al.^7^ utilized the Controlling Nutritional Status (CONUT) score as a measure of nutritional status and observed that it exhibited a more robust and significant association with all-cause mortality than the PNI in patients with ST-segment elevation myocardial infarction (STEMI).

Based on the evidence mentioned above, we propose that PNI could serve as a dependable factor for evaluating and predicting long-term outcomes in patients with ACS. To further explore this potential, we conducted a meta-analysis incorporating updated data from published studies. The primary aim of our research was to investigate the association between PNI and the risk of all-cause mortality in patients undergoing percutaneous coronary intervention (PCI) for ACS. The secondary outcomes included (1) the correlation between a low PNI and the risk of major adverse cardiovascular events (MACEs)/major adverse cardiac and cerebrovascular events (MACCEs), and (2) the relationship between other predictors and the risk of all-cause mortality.

## Methods

### Reporting guideline and registration

Details of the protocol registration are available in the International Prospective Register of Systematic Reviews (PROSPERO) (Registration Number: CRD42023392205). We performed this meta-analysis according to the Preferred Reporting Items for Systematic Reviews and Meta-Analyses (PRISMA) checklist.

### Inclusion and exclusion criteria

The eligibility criteria for the current study were as follows: (1) studies focusing on individuals with various types of ACS [for example, STEMI, or non-ST-segment elevation myocardial infarction (NSTEMI)] undergoing PCI. Patient demographics, comorbidities, and study design (e.g., peer-reviewed randomized controlled trials or observational studies) were not subjected to any restrictions in this meta-analysis; (2) studies investigating the correlation between PNI and all-cause mortality or MACEs/MACCEs with a long-term follow-up; and (3) studies with data available for the calculation of hazard ratio (HR).

We excluded studies that (1) only reported in-hospital outcomes; (2) included patients undergoing cardiac surgery; (3) were presented as case reports, review articles, conference abstracts, or letters; and (4) did not involve PCI.

### Data sources and literature searches

The Embase (Ovid), Medline (Ovid), Cochrane Library, and Google Scholar databases were searched from inception to January 17, 2023, using the following search terms: (“prognostic nutrition index” OR “PNI”) AND (“coronary artery disease” OR “acute coronary syndrome” OR “myocardial infarction” OR “coronary heart disease” OR “unstable angina pectoris”). Details of the search strategies are summarized in Supplementary Table 1. To identify potentially missing articles, we screened the reference lists of the retrieved articles. No restrictions were placed on the publication year, language, or sample size for this meta-analysis. Two independent reviewers performed a two-step selection process to determine article eligibility: (1) initial screening of the titles and abstracts of the articles and (2) full-text review of the potentially eligible articles. All disagreements were resolved by a third reviewer.

### Outcomes and data extraction

The association between a low PNI and the risk of all-cause mortality served as the primary outcome, while the secondary outcomes included (1) the correlation between a low PNI and the risk of MACEs/MACCEs, which was defined based on individual studies, and (2) the relationship between other predictors and the risk of all-cause mortality. The data retrieved from the eligible studies included the first author’s name/published year, country, study design, patient population and number, sex distribution, mean or median age at baseline, PNI cutoff values for defining low or high PNI, and follow-up period. In our data extraction process, for studies that categorized patients into tertiles or quartiles based on PNI, we collected data from the lowest and highest tertiles or quartiles. By focusing on extreme groups, we aimed to minimize the impact of potential confounding variables that could exist in the middle tertile or quartile. For studies that provided both unadjusted and adjusted data, we collected adjusted data for analysis.

### Quality of studies and certainty of evidence

In the current meta-analysis, we used the Newcastle–Ottawa Scale (NOS)^17,18^ to assess the quality of non-randomized studies. The NOS evaluates three key aspects: selection of study groups, comparability of these groups, and ascertainment of exposure and outcome. Each study was assigned a star rating for each category, with higher stars indicating better quality. Each study included in our systematic review was independently assessed by two reviewers using these criteria to evaluate methodological quality. Discrepancies in the quality assessment were resolved through discussion and consensus. As previously reported, a study with an overall NOS score of ≥ 7 was regarded as high-quality^17,18^.

Using the Grading of Recommendations Assessment, Development and Evaluation (GRADE) methodology, the certainty of evidence for each outcome fell into the following four categories: very low, low, moderate, or high. Risk of bias and certainty of evidence assessments were performed by two independent reviewers, with disagreements settled through consultation with a third author.

### Statistical analysis

To assess the agreement between the two independent reviewers, we calculated the kappa statistic, which is a commonly used measure to quantify the inter-rater agreement for categorical data. The kappa statistic ranges from − 1 to 1, with 1 indicating perfect agreement, 0 indicating agreement due to chance, and values of − 1 indicating complete disagreement. A kappa statistic of 0.8 or higher is generally considered to be in good agreement, while a kappa statistic of 0.6 or higher is considered to be fair agreement.

The data analysis was performed using two software tools: Review Manager (RevMan) version 5.4 and the “metaphor” package in R software version 4.2.1. A random-effects model was used for all the analyses. We made a predetermined decision to employ a random-effects model for outcome evaluation, regardless of the presence or absence of statistical heterogeneity, based on the assumption that heterogeneity might exist across the included studies. The association of PNI with long-term outcomes was calculated using the PNI value as a continuous (e.g., per point increase in PNI) or categorical (e.g., low vs. high PNI) variable. If several categories of PNI values were provided in a study, the lowest and highest categories of PNI were used for the risk calculation. To evaluate the degree of heterogeneity among the samples, the I^2^ statistic was applied, with statistical significance set at I^2^ > 50%^19^. When significant heterogeneity was found for the primary outcome, the sources of heterogeneity were identified using meta-regression analysis based on sample size, PNI cut-off value, and length of follow-up. A sensitivity analysis was conducted to confirm the robustness of the outcome by sequentially removing one study at a time. Funnel plots were visually examined to identify the risk of publication bias when more than 10 datasets were included in the pooled analysis^20^. Statistical* p* was set than 0.05.

## Results

### Selection, characteristics, and quality of studies

**Figure 1:**
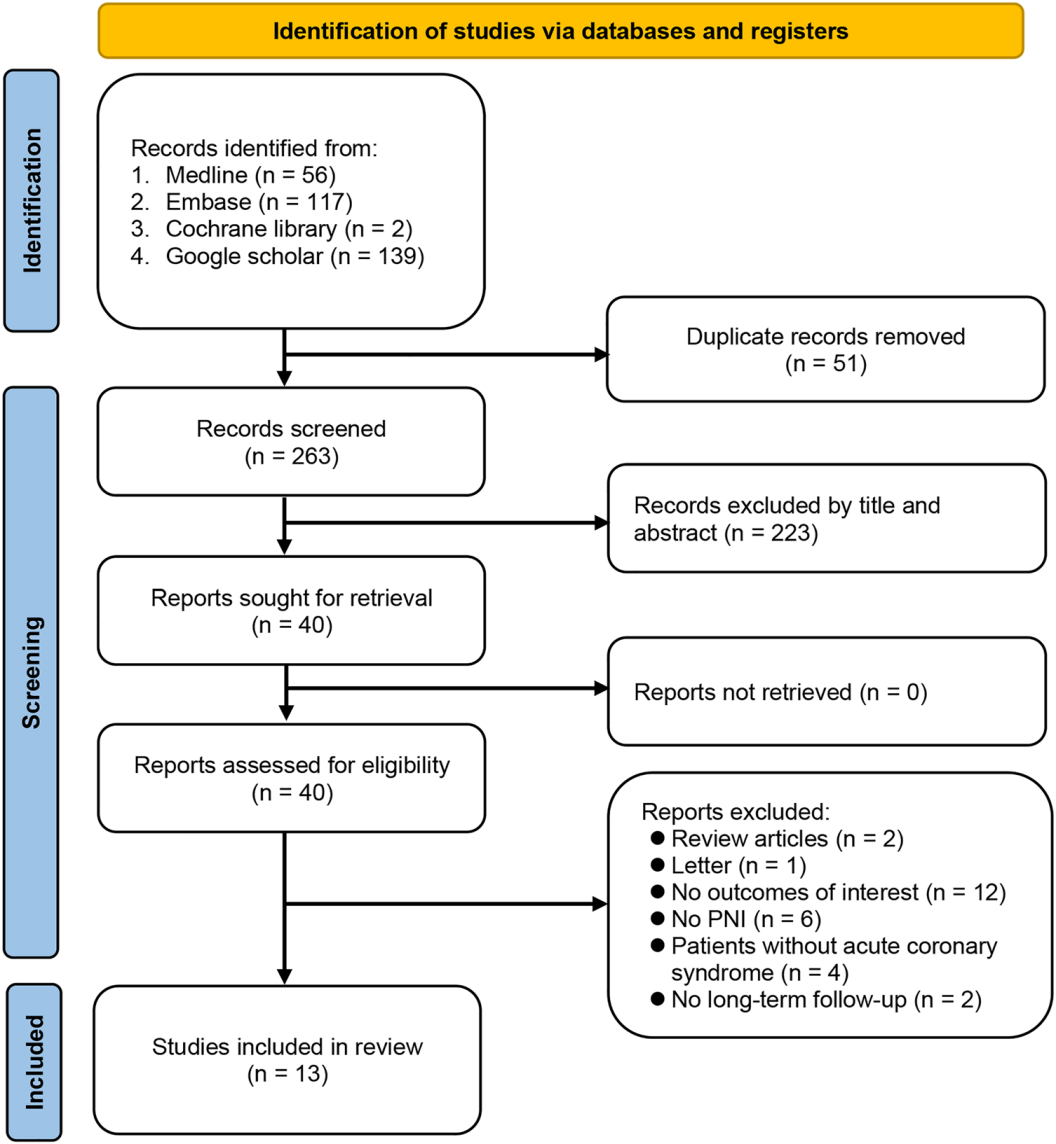
Flowchart of the database search and screening process. PNI: prognostic nutritional index.

Of the 314 potentially relevant records initially identified (Fig. 1), 51 duplicates and 223 irrelevant articles were excluded after screening their titles and abstracts. Of the remaining 40 articles subjected to full-text review, 27 were excluded because of (1) being review articles or letters (n = 3), (2) no outcome of interest (n = 12), (3) no use of PNI as the predictor (n = 6), (4) recruiting patients without ACS (n = 4), and (6) unavailable data on long-term follow-up (n = 2). Finally, 13 retrospective observational studies published between 2017 and 2022 were included in the current meta-analysis and systematic review (Fig. 1)^5–7,16,21–29^. The calculated kappa statistic for the two-step selection process yielded a value of 0.89, indicating a substantial agreement between the two reviewers in selecting studies for inclusion in the systematic review.

**Figure 2:**
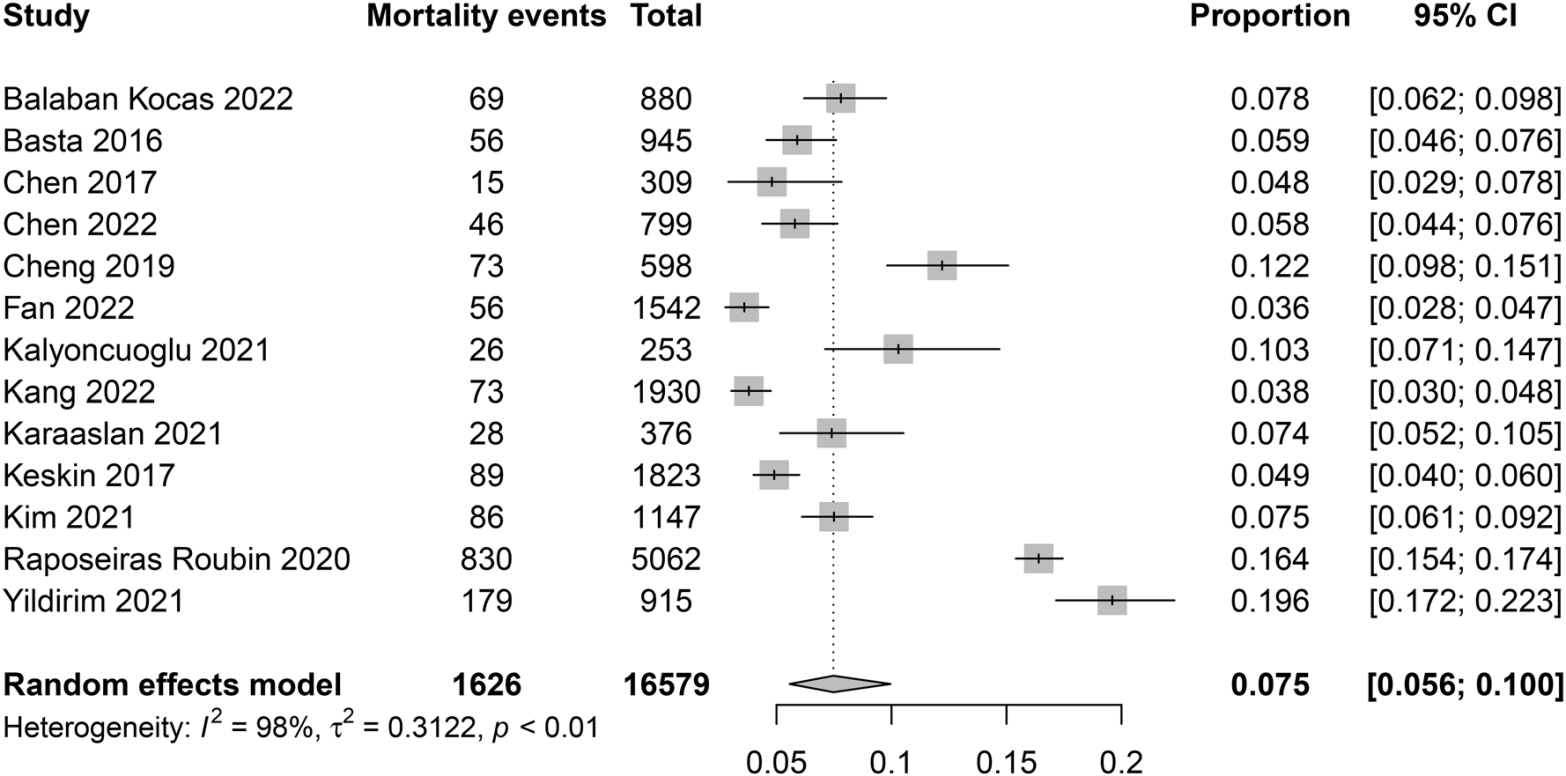
Forest plot showing the pooled incidence of mortality rate (7.5%, 95% confidence interval 5.6–0.0%) in patients receiving percutaneous coronary intervention for acute coronary syndrome.

The characteristics of the 13 eligible studies are summarized in Table 1. The studies were conducted in five countries: Turkey (n = 5)^21,25–27,29^, China (n = 4)^16,22–24^, Korea (n = 2)^5,28^, Italy (n = 1)^7^, and Spain (n = 1)^6^. A total of 16,579 patients were included, with a male proportion of 51.6% and 81%, respectively. Seven of the analyzed studies explicitly stated that they excluded patients with active infections^16,21–23,25–27^. Conversely, in the remaining six studies, there was no explicit mention of the criteria regarding the exclusion of patients with active infection^5–7,24,28,29^. In seven studies, the initial diagnosis of myocardial infarction included STEMI (n = 4)^7,22,27,28^ and NSTEMI (n = 3)^25,26,29^. Another six studies included patients with ACS without mentioning the type of myocardial infarction^5,6,16,21,23,24^. The mortality rate varied widely across the included studies (range: 3.6–19.6%), with a pooled mortality rate of 7.5% (95% CI 5.6–10.0%) (Fig. 2). Table 1 summarize the PNI cut-off values in individual study for calculation of the mortality risk. The follow-up period ranged from 6 months to a maximum of 67.2 months. The quality of studies assessed using NOS is summarized in Table 1; 92.3% of the studies (that is., 12 studies) were considered to have a low risk of bias (≥ 7 stars).

**Table 1:** Characteristics of studies (n = 13).

Author year	Age (years)	Male (%)	AMI	N	Mortality (%)	PNI cut-off values^#^	Outcomes	Follow-up period†	Country	NOS
Balaban Koçaş 2022	59.6	77.9	ACS‡	880	7.8	NA	Mortality/MACEs	12 m	Turkey	5
Basta 2016	65.7	75	STEMI	945	5.9	NA	Mortality	24 m	Italy	7
Chen 2017	58.3	80.9	STEMI	309	4.8	45	Mortality	19.5 m	China	9
Chen 2022	66.3	72.3	ACS‡	799	5.8	NA	Mortality	6 m	China	9
Cheng 2019	64.6	76.3	ACS‡	598	12.2	45	Mortality	14.8 m	China	8
Fan 2022	NA	75	ACS‡	1542	3.6	48.15	MACEs	38.1 m	China	8
Kalyoncuoglu 2021	68.5	71.5	NSTEMI	253	10.3	NA	MACCEs	20.5 m	Turkey	7
Kang 2022	63	67.9	ACS‡	1930	3.8	35 vs. 38	Mortality/MACEs	67.2 m	Korea	8
Karaaslan 2021	73	60	NSTEMI	376	7.4	46	Mortality	11.2 m	Turkey	8
Keskin 2017	58	81	STEMI	1823	4.9	44	Mortality	32.9 m	Turkey	9
Kim 2021	65.6	72.5	STEMI	1147	7.5	50 vs. 56	Mortality	12 m	Korea	9
Raposeiras Roubin 2020	66.2	74.5	ACS‡	5062	16.4	35 vs. 38	Mortality/MACEs	43.2 m	Spain	8
Yildirim 2021	73.1	51.6	NSTEMI	915	19.6	NA	Mortality	64.5 m	Turkey	8

*m* months, *NA* not available, *ACS* acute coronary syndrome, *STEMI* ST-segment elevation myocardial infarction, *NSTEMI* non-ST-segment elevation myocardial infarction.

^†^Mean or median; *MACEs* major adverse cardiovascular events, *MACCEs* major adverse cardiac and cerebrovascular events, *AMI* acute myocardial infarction.

^‡^Patients with ST-elevation myocardial infarction, non-ST elevation myocardial infarction, or unstable angina.

^#^PNI cut-off values for mortality risk.

### Study outcomes

#### Primary outcome: risk of all-cause mortality

**Figure 3:**
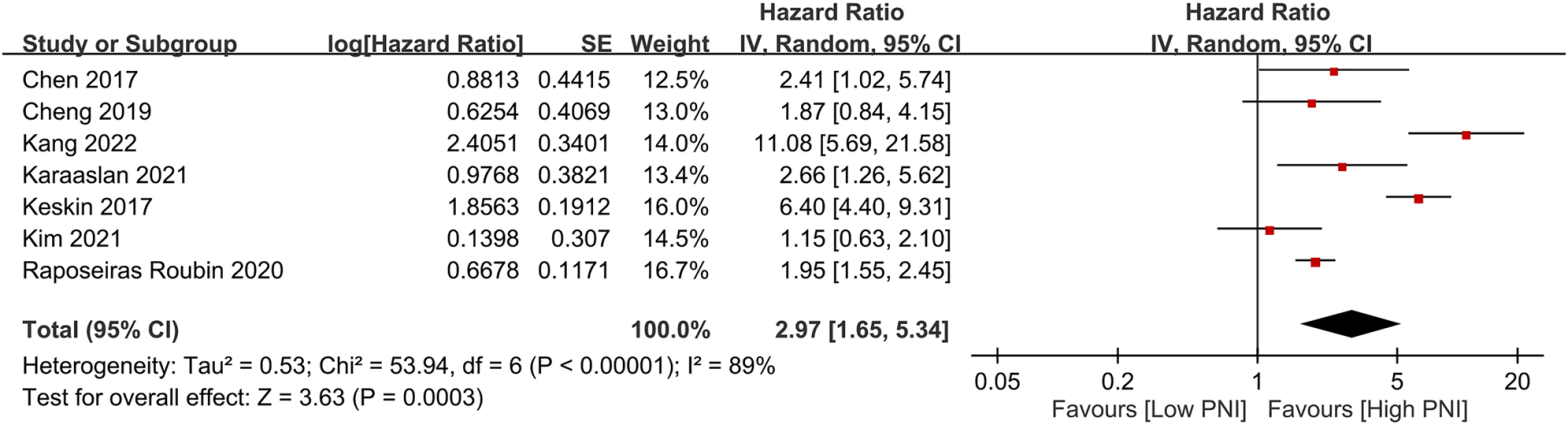
Forest plot comparing the risk of all-cause mortality between patients with a low and those with a high prognostic nutritional index (PNI). *IV* inverse variance; *CI* confidence interval; *SE* standard error.

**Figure 4:**
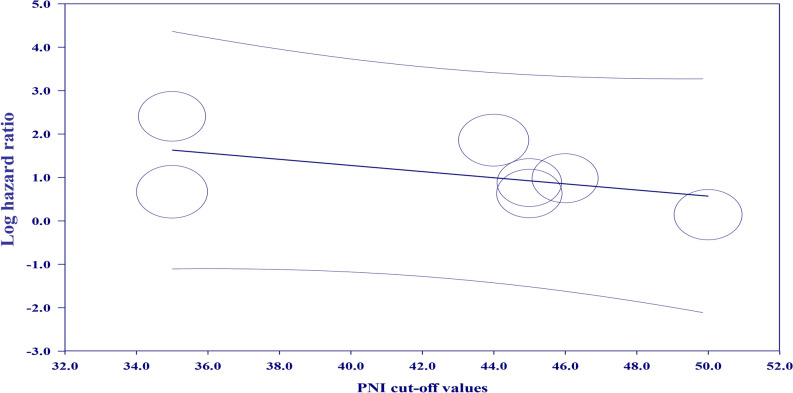
Meta-regression plot for the risk of all-cause mortality by cut-off values of prognostic nutritional index (PNI), showing non-significant coefficient for the PNI cut-off values (− 0.07; 95% confidence interval [CI] − 0.21 to 0.06, *p* = 0.3).

The association of PNI as a categorical variable (i.e., low vs. high PNI) with the risk of all-cause mortality was available in seven studies^5,6,22,23,26–28^ (i.e., primary outcome). For patients with a low PNI, the pooled HR of all-cause mortality was 2.97 (95% CI 1.65 to 5.34, *p* = 0.0003, I^2^ = 89%, seven studies, 11,245 patients) (Fig. 3)^5,6,22,23,26–28^. Sensitivity analysis revealed consistent results. The certainty of the evidence for this outcome was very low. Details regarding the certainty of the evidence are summarized in Supplemental Table 2. Meta-regression analysis demonstrated no impact of the included covariates, including PNI cut-off value (*p* = 0.3) (Fig. 4), follow-up period (*p* = 0.08) (Supplemental Fig. 1), and sample size (*p* = 0.95) (Supplemental Fig. 2), on the relationship between a low PNI and the risk of all-cause mortality.

**Figure 5:**
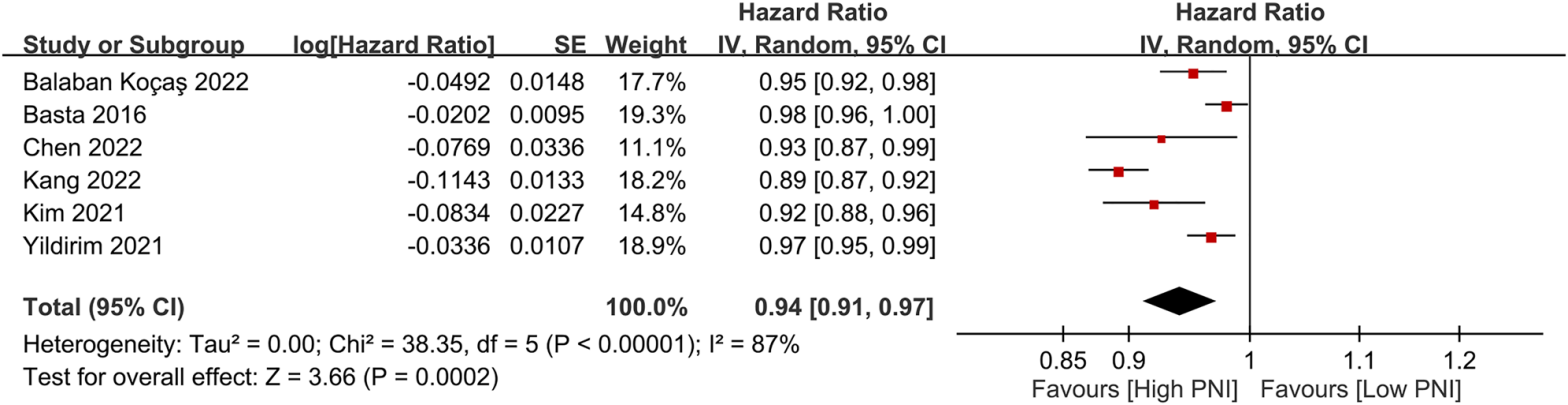
Forest plot showing reduction in the risk of all-cause mortality with per unit increase in prognostic nutritional index (PNI). *IV* inverse variance; *CI* confidence interval, *SE* standard error.

Similarly, six studies focusing on PNI as a continuous variable also demonstrated a reduced risk of all-cause mortality with each unit increase in PNI (HR 0.94, 95% CI 0.91 to 0.97, *p* = 0.0002, I^2^ = 87%, six studies, 6616 patients, certainty of evidence: very low) (Fig. 5)^5,7,16,21,28,29^. This finding was consistent with that of the sensitivity analysis.

#### Secondary outcome: risk of MACEs/MACCEs

**Figure 6:**
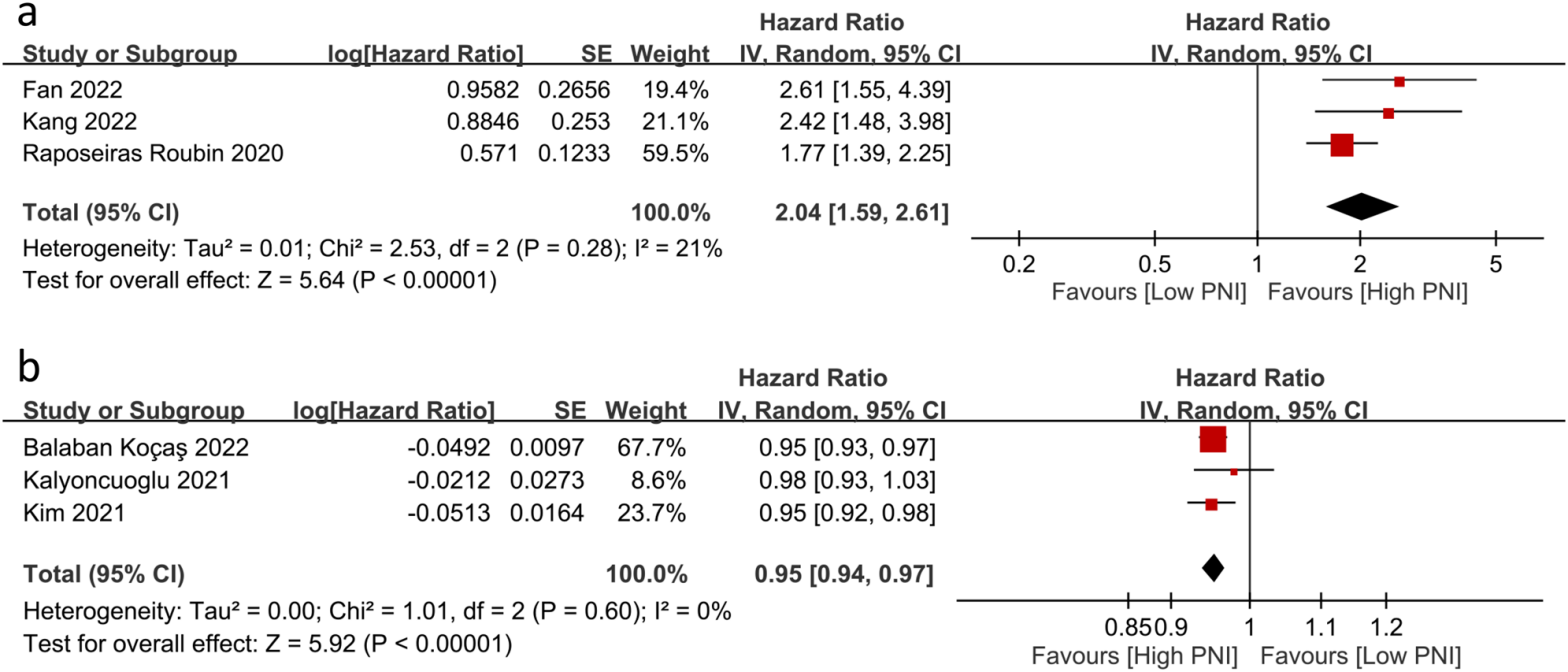
Forest plots showing an association of the risks of major adverse cardiovascular events (MACEs)/major adverse cardiac and cerebrovascular events (MACCEs) with prognostic nutritional index (PNI) as a (**a**) categorical or (**b**) continuous variable. *IV* inverse variance; *CI* confidence interval, *SE* standard error.

Six studies reported an association between the risk of MACEs (n = 4)^5,6,21,24^ or MACCEs (n = 1)^25^ with PNI as a categorical variable (i.e., low vs. high) (three studies)^5,6,24^ or as a continuous variable (three studies)^21,25,28^. Using PNI as a categorical variable (i.e., low vs. high), a meta-analysis showed a higher risk of MACEs in patients with a low PNI (HR 2.04, 95% CI 1.59 to 2.61, *p* < 0.00001, I^2^ = 21%, 8534 patients, certainty of evidence: low) (Fig. 6a)^5,6,24^. The sensitivity analysis revealed a consistent finding, suggesting the robustness of the evidence. In concert with this finding, analysis of the three studies that used PNI as a continuous variable for MACE/MACCE risk prediction also demonstrated an inverse relationship between the value of PNI and risk of MACE/MACCE (HR 0.95, 95% CI 0.94 to 0.97, *p* < 0.00001, I^2^ = 0%; three studies, 2280 patients; certainty of evidence: low) (Fig. 6b)^21,25,28^.

#### Secondary outcomes: other predictors of all-cause mortality

Other predictors of all-cause mortality are summarized in Table 2. In brief, advanced age (HR 1.04, 95% CI 1.01 to 1.07), diabetes mellitus (HR = 1.57, 95% CI 1.08 to 2.3), and high GRACE risk scores (HR 1.02, 95% CI 1.02 to 1.03) were associated with a high risk of all-cause mortality. In contrast, a high body mass index (HR 0.87, 95% CI 0.76–0.98) was associated with a low risk of all-cause mortality.

**Table 2:** Predictors of all-cause mortality in patients undergoing percutaneous coronary intervention for acute coronary syndrome.

Parameters	Studies (n)	Number of patients	Effect estimate Hazard ratio [95%]	*P* value	Heterogeneity (%)
Age (years)	4	3049	1.04 [1.01, 1.07]	0.002	76
Male gender	2	1679	1.29 [0.80, 2.09]	0.29	0
Body mass index (kg/m^2^)	2	1513	0.87 [0.76, 0.98]	0.02	75
Hypertension	2	1679	1.41 [0.96, 2.06]	0.08	0
Diabetes mellitus	2	1679	1.57 [1.08, 2.30]	0.02	0
Previous MI	3	2055	1.44 [0.86, 2.40]	0.16	53
Creatinine (μmol/L)	2	1189	1.12 [0.89, 1.41]	0.32	96
Ejection fraction (%)	5	3078	0.98 [0.91, 1.05]	0.52	96
Total cholesterol (mmol/L)	3	1706	1.01 [0.98, 1.04]	0.4	0
Triglyceride (mmol/L)	2	1397	0.93 [0.73, 1.17]	0.53	52
LDL (mmol/L)	2	1773	1.00 [0.99, 1.01]	0.69	0
GRACE risk score	3	1706	1.02 [1.02, 1.03]	< 0.00001	6

*MI* myocardial infarction, *LDL* low-density lipoprotein, *GRACE* global registry of acute coronary events.

## Discussion

This meta-analysis, which involved 13 retrospective studies including 16,579 patients receiving PCI for ACS followed from 12 to 67.2 months, showed a positive correlation between a low PNI, either as a categorical or continuous variable, and an increased risk of all-cause mortality. Likewise, our analysis of six studies focusing on MACEs/MACCEs revealed increased risks of these long-term complications in patients with a low PNI. The robustness of the evidence was supported by consistent results of the sensitivity analyses. In addition, our study demonstrated a significant association between other risk factors, namely advanced age, diabetes, and high GRACE risk scores, and elevated long-term mortality in patients with ACS. Interestingly, high body mass index was associated with a reduced risk of all-cause mortality. Taken together, our study showed that poor nutritional status, as reflected by a low PNI, correlated with an increased risk of mortality and long-term complications in patients receiving PCI for ACS.

An analysis from the Korea Acute Myocardial Infarction Registry (KAMIR) reported an independent negative association between body mass index and risk of long-term cardiovascular events in patients with ACS^30^. Consistently, the current study demonstrated a negative correlation between BMI and mortality risk. Therefore, our findings provide robust evidence supporting the significance of a low PNI as a specific red flag for predicting poor outcomes in patients with ACS after coronary interventions. Prior studies have shown that the pathophysiological mechanisms by which nutrition is linked to ACS are complex and multi-faceted^6,31,32^. For instance, chronic low-grade inflammation triggered by malnutrition can exacerbate the development of coronary artery disease^32,33^. In addition, malnutrition can increase oxidative stress in the body, thereby contributing to the development of ACS^33,34^. Oxidative stress, the accumulation of reactive oxygen species, causes damage to cells and tissues, including the coronary arteries^34^. Furthermore, an inadequate supply of nutrients, such as glucose and amino acids, can lead to cardiac muscle dysfunction^31,35^. Previous investigations have identified poor nutrition, which has been frequently ignored as an important risk factor for negative cardiac remodeling and endothelial dysfunction^36–38^. Moreover, poor nutritional status can result in muscle wasting and impaired immune function, which negatively impacts recovery from ACS and increases the risk of complications such as infections^39^. Lastly, malnutrition may predispose to disturbances in lipid metabolism and increase the levels of certain lipids [e.g., such as low-density lipoprotein (LDL) cholesterol], thereby affecting the equilibrium among different lipids in the circulatory system in favor of cardiovascular diseases^40^. Therefore, malnutrition can significantly impact the development and progression of ACS. Understanding the mechanisms by which malnutrition contributes to ACS is important for implementing appropriate preventive and therapeutic strategies.

A previous study reported that lower lymphocyte count was associated with an elevated risk of long-term mortality and cardiovascular readmissions^41^. Considering that the PNI is computed by summing the serum albumin (g/L) and five times the total lymphocyte count (10^9^/L), it is possible that the observed association between low PNI values and increased mortality rates in the present meta-analysis could, in part, be attributable to a diminished lymphocyte count. The comparative effectiveness of PNI in predicting mortality in patients with ACS remains to be fully elucidated when compared with other nutritional indices. For instance, Basta et al. reported that the CONUT index, which includes serum albumin, total cholesterol levels, and total lymphocyte count, was superior to the PNI in predicting all-cause death in patients with STEMI^7^. Additionally, the proportion of patients at risk of malnutrition, as evaluated using the CONUT index, was higher than that assessed using the PNI index (60% vs. 52%)^7^. Further large-scale studies are required to comprehensively address these important issues.

As a previous study demonstrated an association between low serum albumin levels and adverse outcomes in patients with STEMI^42^, it would be of interest to investigate the individual performance of albumin as a prognostic indicator and compare it with that of the PNI. A meta-analysis, which included eight studies comprising 21,667 ACS patients, revealed that ACS individuals with low serum albumin levels were at an elevated risk of long-term mortality (risk ratio:1.75)^43^. In the current meta-analysis, we found that a low PNI was associated with significantly increased long-term mortality risk (hazard ratio: 2.97). While it may not be entirely rational to directly compare the predictive strengths of the two parameters (i.e., PNI and albumin), these findings suggested that the use of PNI to predict long-term outcome may be favorable.

In addition to PNI, risk assessment scores, including the Geriatric Nutritional Risk Index (GNRI), CONUT score, Nutritional Risk Index (NRI), Subjective Global Assessment (SGA), and Nutritional Risk Screening, have been introduced to predict the outcomes of patients diagnosed with CAD^28,33,44^. Although GRACE risk scores that consist of hemodynamic parameters and electrocardiographic and laboratory information have been used and validated for the prediction of both all-cause mortality and MACEs in patients with acute myocardial infarction^6,45^, nutritional status was not taken into consideration. An observational study has shown enhanced accuracy in predicting long-term outcomes in individuals with ACS by combining three nutritional scores, namely the CONUT, NRI, and PNI scores, with the GRACE risk score^6^. Similarly, another study demonstrated that a combination of PNI and GRACE score significantly improved the prediction of long-term mortality in patients undergoing coronary interventions for ACS^16^. However, the results of most studies have been derived from a limited number of participants with heterogeneous characteristics^6,46^. Therefore, although patients with CAD in a poor nutritional status might generally have a worse prognosis^29^, there is no pooled evidence showing the sensitivity of these indices for the prediction of long-term outcomes in this patient population. Our meta-analysis is the first to investigate the association of a low PNI with the risk of all-cause mortality as well as MACEs/MACCEs.

The current meta-analysis has several limitations that deserve consideration. First, our analysis was based solely on observational studies, which inherently carry a risk of bias and confounding factors. The lack of randomized controlled trials may limit the strength of our conclusions and warrants caution when interpreting the results. Second, the severity of myocardial injury varied among individual studies, with four and three focusing on STEMI and NSTEMI, respectively, while all the others did not mention the type of MI in their participants. Because of the limited number of studies, subgroup analysis was not conducted to investigate the relationship between low PNI and mortality based on the severity of myocardial injury. Third, despite the identification of advanced age, diabetes, and high GRACE risk scores at baseline as risk factors for long-term mortality in patients with ACS in the current meta-analysis, their predictive values may be weak because of the limited number of studies that provide relevant data for the estimation of effect size. Fourth, while we attempted to address potential confounders by collecting adjusted data from studies that provided both unadjusted and adjusted estimates, residual confounding may still exist because of unmeasured or unknown variables across the included studies. For example, other confounding factors, such as smoking, family, and drug history were not included. Fifth, active infection may have a potential influence on PNI, albumin and lymphocyte levels. In the current meta-analysis, six studies did not explicitly mention the exclusion of patients with active infections. Consequently, there is uncertainty regarding whether patients with infections were included in their study cohorts, which may have introduced potential confounding factors. Finally, although combining the conventional risk scores (e.g., GRACE) and malnutrition may help differentiate ACS patients who are at high risk of mortality from MACEs/MACCEs, the feasibility of such a combination approach was not evaluated in the current meta-analysis because of a lack of relevant information in individual studies.

## Conclusions

This meta-analysis showed that patients with a low PNI who underwent coronary interventions had an elevated risk of long-term mortality and MACEs/MACCEs. In addition, other risk factors, namely advanced age, diabetes, and high GRACE risk scores, were correlated with elevated long-term mortality in patients with ACS, while a high body mass index was associated with a reduced risk of all-cause mortality. The results of our meta-analysis provide clinical practitioners with an additional tool for assessing the risks of patients with ACS, going beyond conventional cardiovascular risk factors. PNI showed potential as a straightforward and readily available prognostic indicator, facilitating risk stratification and guiding personalized patient management strategies. To reinforce the evidence base and achieve a more comprehensive understanding of the role of PNI in ACS outcomes, further studies, including randomized controlled trials, are required.

### Supplementary Information

Supplementary Information.

## Data Availability

All data related to the present systematic review and meta-analysis are available from the corresponding author upon reasonable request.
