# Supplementary material for: Association of prognostic nutritional index with long-term mortality in patients receiving percutaneous coronary intervention for acute coronary syndrome: a meta-analysis
Source: Sci Rep. 2023 Aug 11;13:13102. doi: 10.1038/s41598-023-40312-4 (PMC10421894; doi:10.1038/s41598-023-40312-4)

**Supplemental Table 1.** Search strategies for four databases

| Database |  | Search syntax |
| --- | --- | --- |
| **PubMed/MEDLINE (Ovid)** | #1 | ("PCI" or "Coronary Angiograph*" or "coronary artery disease" or "Percutaneous Coronary Intervention" or "Myocardial infarction" or "MI" or “unstable angina pectoris”).mp. |
|  | #2 | exp "Percutaneous Coronary Intervention"/ or exp "coronary artery disease"/ or exp "Coronary Angiography"/ |
|  | #3 | ("Prognostic nutritional index" or "Prognostic Nutritional Indices" or "PNI").mp. |
|  | #4 | (1 or 2) and 3 |
| **Embase (Ovid)** | #1 | ("PCI" or "Coronary Angiograph*" or "coronary artery disease" or "Percutaneous Coronary Intervention" or "Myocardial infarction" or "MI" or “unstable angina pectoris”).mp. |
|  | #2 | exp "Percutaneous Coronary Intervention"/ or exp "coronary artery disease"/ or exp "Coronary Angiography"/ |
|  | #3 | ("Prognostic nutritional index" or "Prognostic Nutritional Indices" or "PNI").mp. |
|  | #4 | (1 or 2) and 3 |
| **Cochrane CENTRAL** | #1 | ("PCI" or "Coronary Angiograph*" or "coronary artery disease" or "Percutaneous Coronary Intervention" or "Myocardial infarction" or "MI" or “unstable angina pectoris”): ti,ab,kw |
|  | #2 | [mh "Percutaneous Coronary Intervention"] OR [mh "coronary artery disease"] OR [mh "Coronary Angiography"] |
|  | #3 | ("Prognostic nutritional index" or "Prognostic Nutritional Indices" or "PNI"): ti,ab,kw |
|  | #4 | (#1 OR #2) AND #3 |
| **Google scholar** |  | A hand-search strategy was used to identify the related studies, followed by using a forward snowballing strategy to retrieve all potentially eligible articles. |

**Supplemental Table 2.** Certainty of evidence

| Outcomes | Relative effect (95% CI) | № of participants  (studies) | Certainty of the evidence (GRADE) | Comments |
| --- | --- | --- | --- | --- |
| Risk of all-cause mortality (PNI as a categorical variable) | HR: 2.97  (1.65 to 5.34) | 11245 (7 studies) | ⨁◯◯◯ Very Low | wide 95% CI, high heterogeneity |
| Risk of all-cause mortality (PNI as a continuous variable) | HR: 0.94  (0.91 to 0.97) ^‡^ | 6616  (6 studies) | ⨁◯◯◯ Very Low | High heterogeneity |
| Risk of MACEs/MACCEs (PNI as a categorical variable) | HR: 2.04  (1.59 to 2.61) | 8534  (3 studies) | ⨁⨁◯◯ Low | - |
| Risk of MACEs/MACCEs (PNI as a continuous variable) | HR: 0.95  (0.94 to 0.97) ^†^ | 2280  (3 studies) | ⨁⨁◯◯ Low | - |

‡a lower risk of all-cause mortality with per point increase in PNI; †a low risk of MACEs/MACCEs with per point increase in PNI; MACEs: major adverse cardiovascular events; MACCEs: major adverse cardiac and cerebrovascular events PNI: prognostic nutritional index

**GRADE Working Group grades of evidence:**

1. High certainty: We are very confident that the true effect lies close to that of the estimate of the effect
2. Moderate certainty: We are moderately confident in the effect estimate: The true effect is likely to be close to the estimate of the effect, but there is a possibility that it is substantially different
3. Low certainty: Our confidence in the effect estimate is limited: The true effect may be substantially different from the estimate of the effect
4. Very low certainty: We have very little confidence in the effect estimate: The true effect is likely to be substantially different from the estimate of effect

**Supplemental Figure 1.** Meta-regression plot for risk of all-cause mortality by follow-up period. The meta-regression coefficient for the follow-up period was not statistically significant (0.028; 95% confidence interval [CI], −0.004 to 0.06, P = 0.08).


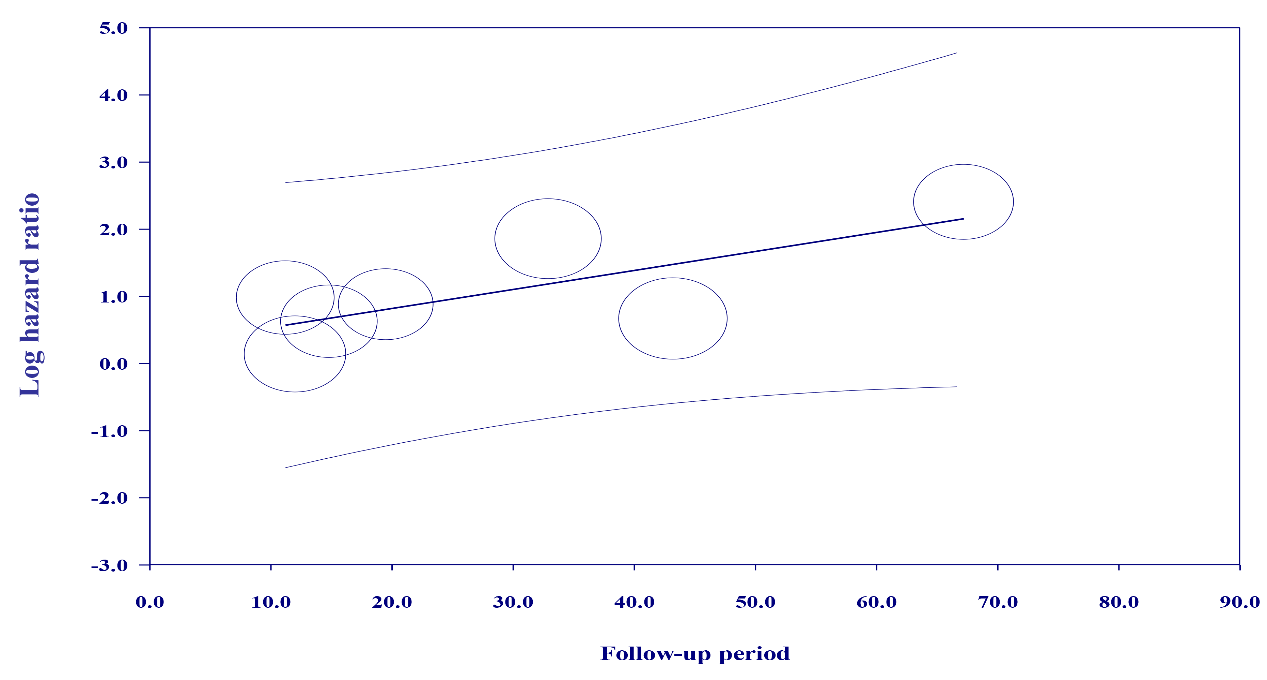


**Supplemental Figure 2.** Meta-regression plot for risk of all-cause mortality by sample size. The meta-regression coefficient for the sample size was not statistically significant (0.000; 95% confidence interval [CI], −0.0004 to 0.0005, P = 0.95).


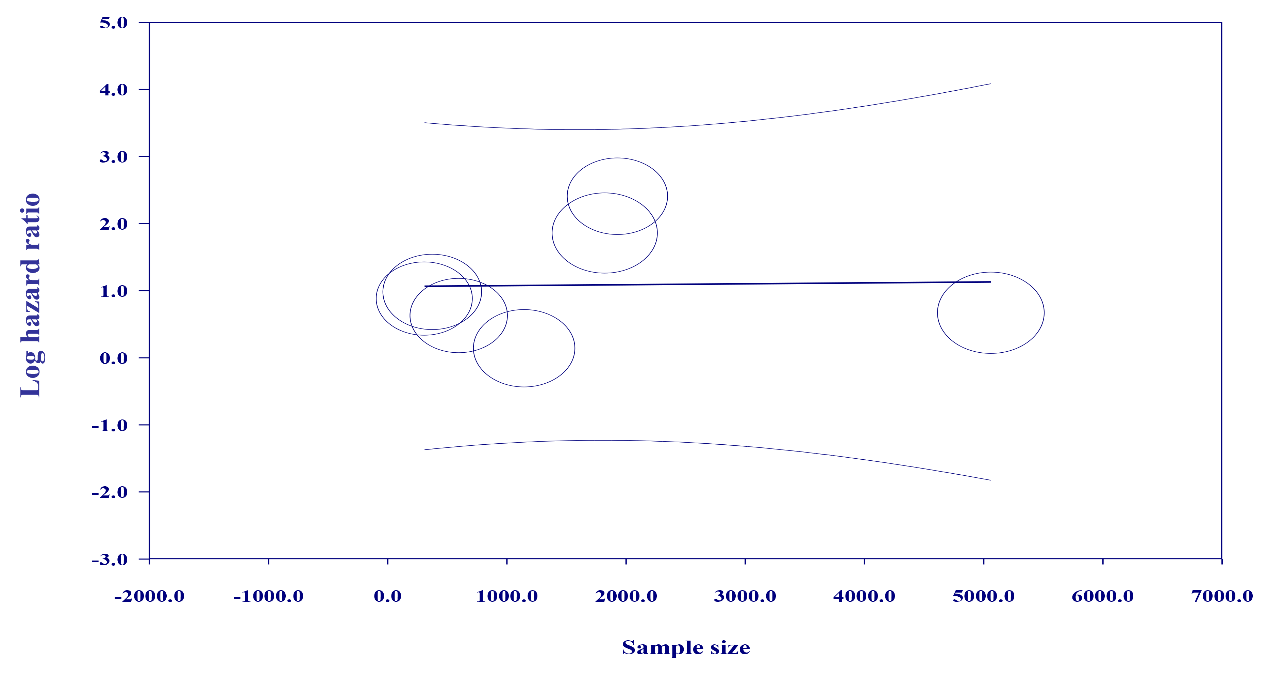

Supplement: Supplementary file 1 — Supplementary Information. [file 41598_2023_40312_MOESM1_ESM.docx]
